# Supplementary material for: MyoD1 localization at the nuclear periphery is mediated by association of WFS1 with active enhancers
Source: Nat Commun. 2025 Mar 17;16:2614. doi: 10.1038/s41467-025-57758-x (PMC11914251; doi:10.1038/s41467-025-57758-x)
Supplement: Supplementary file 1 — Supplementary Information [file 41467_2025_57758_MOESM1_ESM.pdf]

Supplementary Information for

MyoD1 localization at the nuclear periphery is mediated by association of WFS1 with active enhancers

Georgiou *et al.*

Correspondence to:

nana.naetar@meduniwien.ac.at

roland.foisner@meduniwien.ac.at

This document includes:

- **Supplementary Figures 1 to 9**
- **Supplementary Methods**
- **Supplementary Tables 1 to 8**
- **Supplementary References**

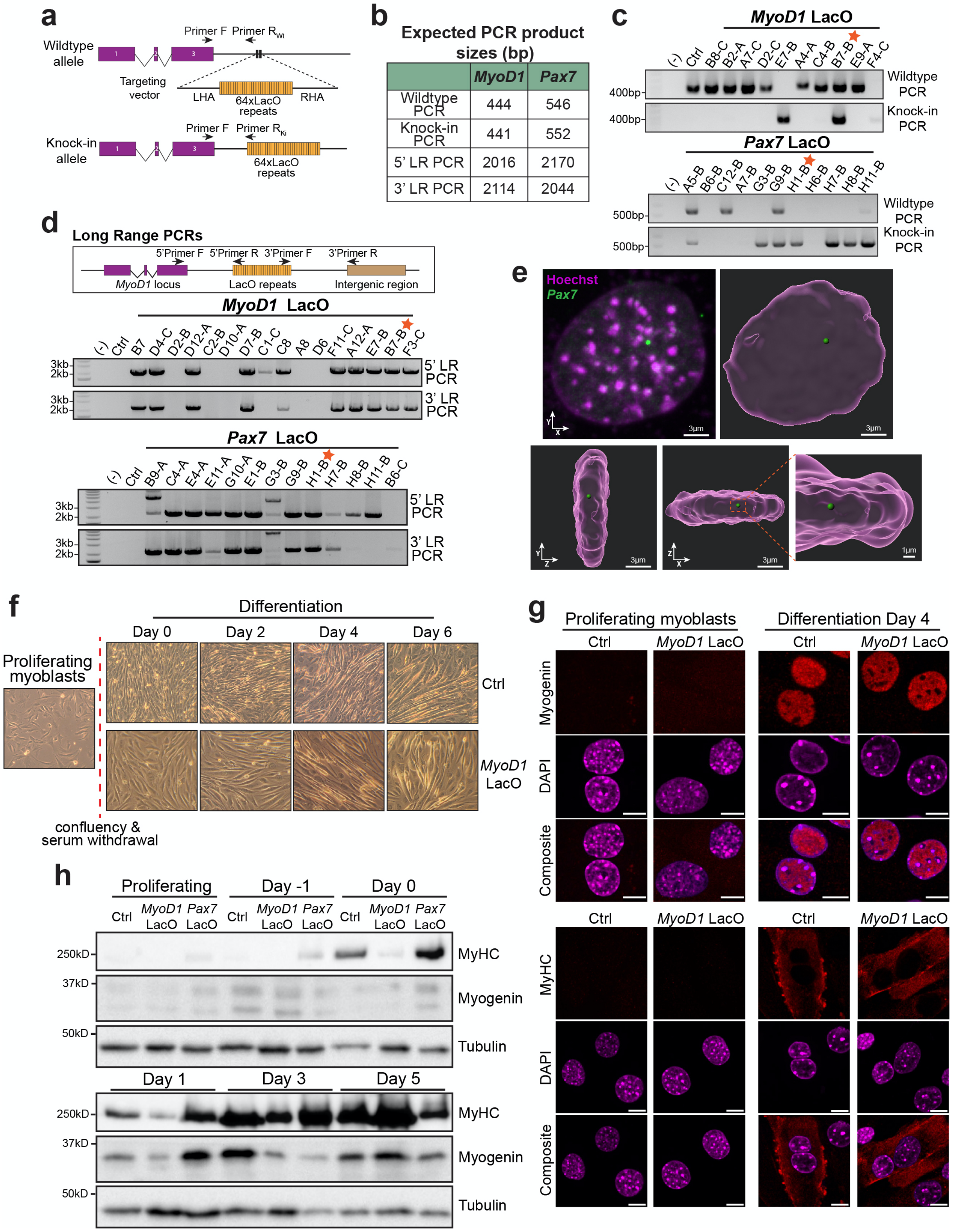

Supplementary Figure 1. Generation of *MyoD1* and *Pax7* reporter cell lines using CRISPR/Cas9. (a) Schematic view of 3 exons (purple boxes), introns (angled lines) and adjacent intergenic region (line) of the mouse *MyoD1* gene locus. Position of the sgRNA target sequence 1 kb downstream of the *MyoD1* gene is marked with two perpendicular lines (top). The targeting vector serving as homology-directed

repair template after Cas9-induced double strand break is indicated (middle). Bottom: Knock-in allele following the successful integration of 64 LacO (Lac operon) repeats. LHA: Left Homology Arm; RHA: Right Homology Arm. Black arrows indicate primer pairs used for genotyping PCRs, F: Forward; R_{Wt}: Reverse wildtype; R_{Ki}: Reverse knock-in. **(b)** Table summarizes expected product sizes of genotyping and long range (LR) PCRs. **(c)** Genomic DNA was isolated from *MyoD1* (top) or *Pax7* (bottom) LacO-tagged single-cell clones. Clonal genotype was determined by PCR analysis using primer pairs indicated in (a). Orange stars denote clones used for further analyses. **(d)** Clones with a knock-in allele were further analyzed for correct targeting construct integration by long range (LR) PCR. Black arrows in schematic drawing indicate the position of primer pairs used for 5' and 3' LR PCRs. Orange stars indicate *MyoD1* and *Pax7* reporter clonal cell lines used for further analyses. F: Forward; R: Reverse; LacO. **(e)** Representative 3D reconstructed z stack of images showing intranuclear position of the *Pax7* locus in proliferating myoblasts. Scale bars are indicated on images. See also Supplementary Movie 2. **(f-h)** Differentiation potential of *MyoD1* C2C12 reporter cell line was assessed. Cells were grown on collagen-coated dishes or slides and, upon reaching confluency, differentiation was induced by serum withdrawal. Proliferating myoblasts and differentiating cells at various time points (as marked on panels) of indicated genotypes were processed for: **(f)** light-microscopy, **(g)** confocal immunofluorescence microscopy and **(h)** immunoblot analysis using antibodies against myogenin and myosin heavy chain (MyHC). LacO: Lac operon; Ctrl: control. Images in (f) were obtained using a light microscope at 100x magnification (10x objective plus 10x magnification of the ocular). Scale bars, 10 µm. Images in (e) processed with Imaris and in (g) with Fiji. Immunofluorescence and immunoblot experiments shown as representative examples were repeated independently two times with similar results. Source data are provided as a Source Data file.

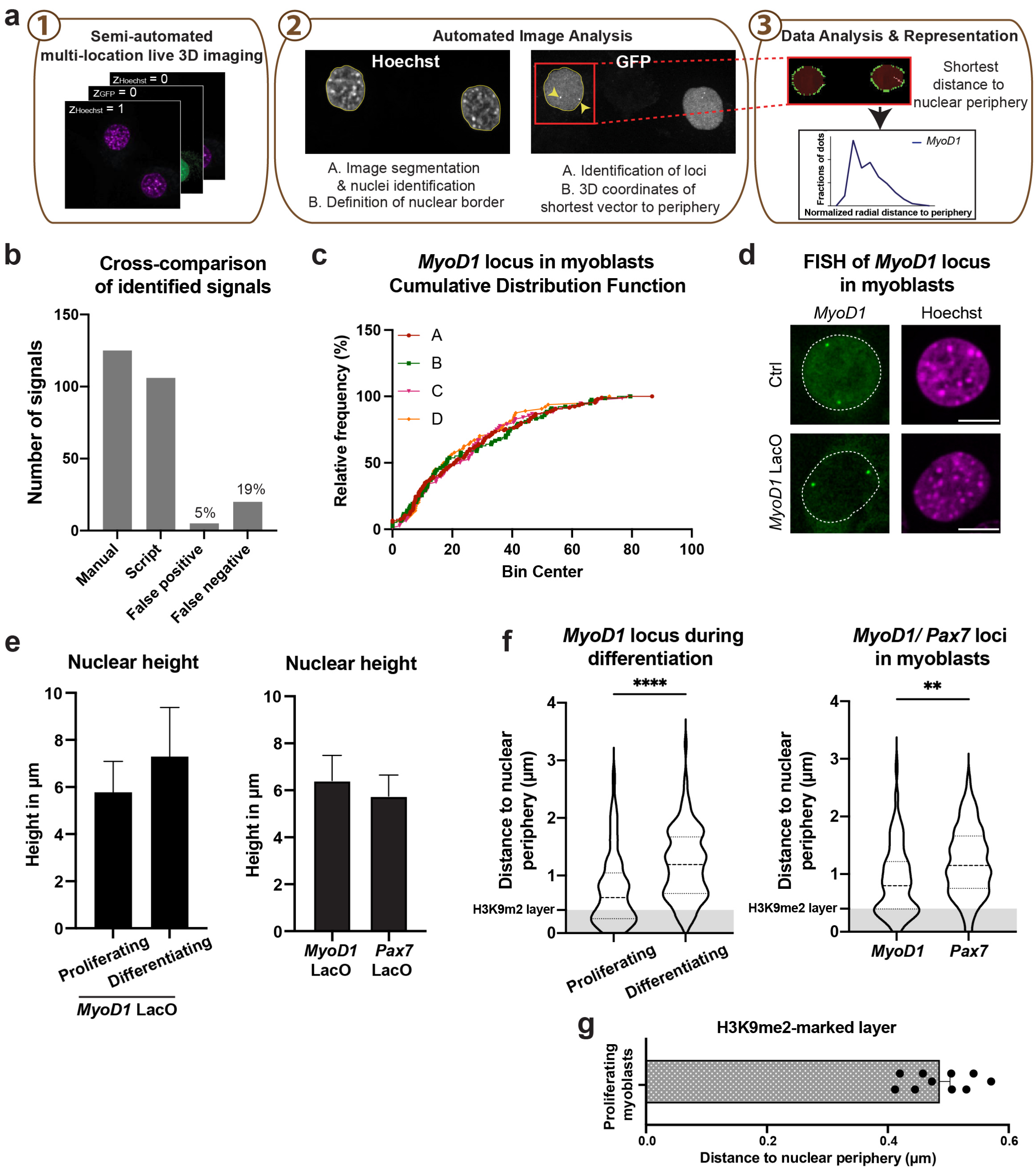

Supplementary Figure 2. Evaluation of automated analyses of intranuclear localization of genomic loci. (a) Schematic overview of workflow for analyses of intranuclear position of gene loci. LacO-tagged reporter cell lines are cultured on collagen-coated imaging slides and DNA is stained with Hoechst shortly prior to image acquisition using semi-automated multi-location three-dimensional live imaging microscopy. Automated image analysis by a custom-made Python plug-in for Fiji entails segmentation and definition of the nuclear border based on DNA staining, followed by detection of the loci and measurement of the shortest distance of the gene loci (GFP) to the nuclear border in 3D. Normalized radial distances to the periphery are calculated and plotted as distributions in density curves and/or violin plots. **(b-c)** Accuracy and reproducibility of automatic signal detection is confirmed by cross-

comparison to manually detected loci (b) and comparison of repeated measurements (c). **(b)** Signals were identified manually and using the Python plug-in script in proliferating myoblasts (n_{manual} = 125, $n_{plug\text{-}in}$ = 106, 39 fields), and false positive and negative rates of the automated analyses were calculated. **(c)** Semi-automatic microscopic detection and automatic analysis was performed on four samples of *MyoD1* LacO-tagged myoblasts and cumulative distribution functions were plotted for each replicate. CDFs represent n_A = 141, n_B = 128, n_C = 82, n_D = 88 (p value> 0.05, two-sample KS test). **(d)** Representative confocal images of fluorescence in-situ hybridization (FISH) using a GFP labeled BAC probe for the *MyoD1* gene in control (untagged) and LacO-tagged myoblasts. Scale bars, 10 µm. FISH experiment shown as representative example was repeated independently two times with similar results. LacO: Lac operon; Ctrl: control. **(e)** Height of nuclei in indicated cell-types was determined and plotted as bar graphs representing the mean ± SD. LacO: Lac operon. **(f)** Distance of genetic loci to the nuclear periphery is displayed as absolute values in violin plots. H3K9me2-positive heterochromatin layer is indicated as gray-shaded horizontal bar. Left: n_{prolif}= 106, n_{diff}= 120 ****p= 1.7 x 10^{-5}. Right: n_{MyoD1}= 208, n_{Pax7}= 79, **p= 0.0025. p values were determined using two-sample, two-tailed KS test and corrected for multiple testing using Hochberg method. **(g)** Width of H3K9me2-positive heterochromatic layer measured in *MyoD1* LacO-tagged proliferating myoblasts. Bar graph shows the mean width ± SEM of 100 locations. Images processed with Fiji. Source data are provided as a Source Data file.

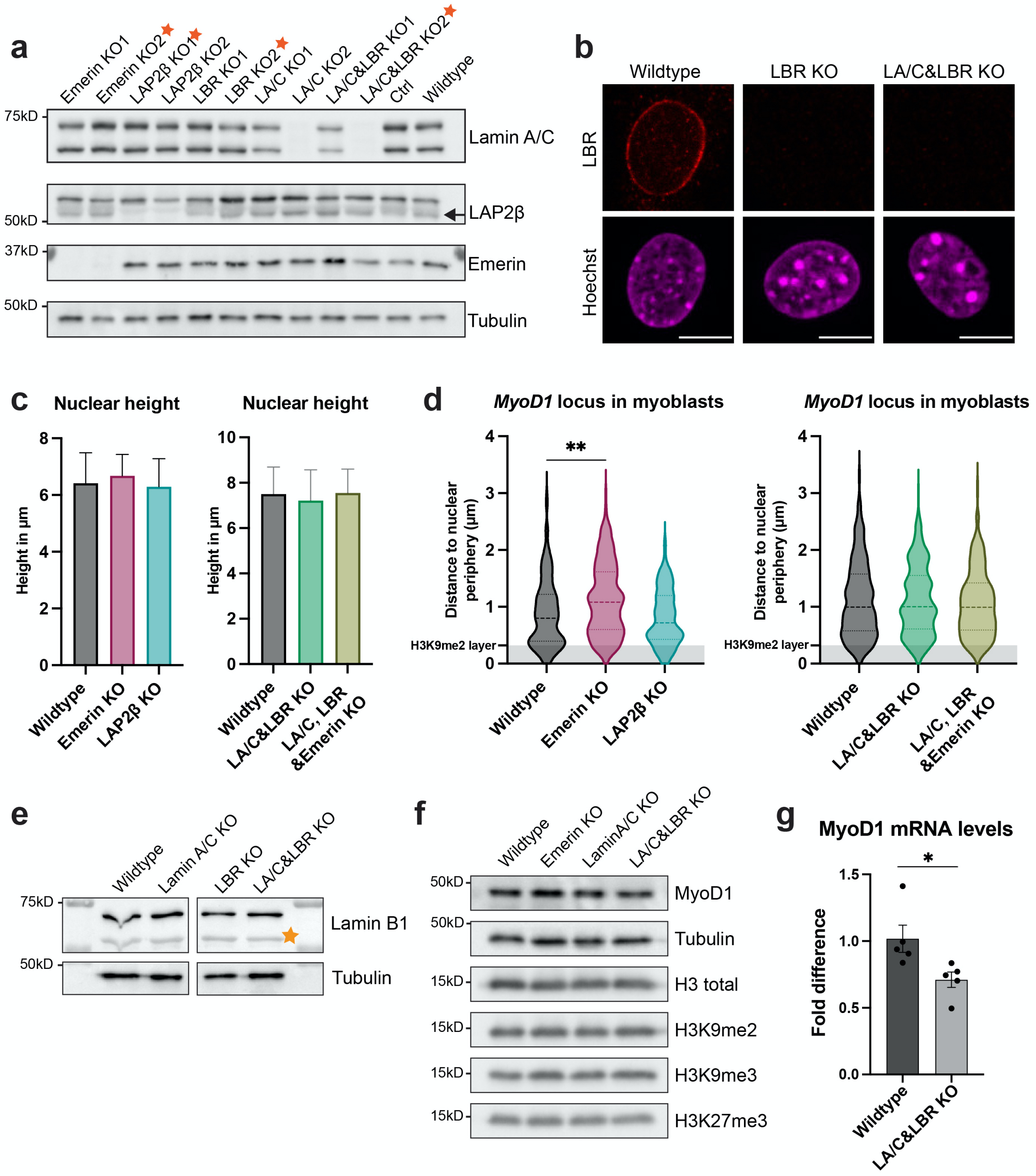

Supplementary Figure 3. Analyses of *MyoD1* LacO reporter myoblasts following knockout of known heterochromatin anchors at the nuclear periphery. (a, b) Depletion of targeted proteins in single cell clones was tested either by **(a)** Western blotting using antibodies to the indicated antigens (lamin A/C, LAP2β and emerin) or **(b)** immunofluorescence staining for LBR. Untagged cells (Ctrl) and cells carrying the LacO tag downstream of the *MyoD1* gene (wildtype) were used as controls. Orange stars mark single cell clones selected for further experiments, black arrow marks the specific LAP2β band. Scale bars, 10 µm. LA/C: lamin A/C, Emd: emerin. **(c)** Nuclear height of cells with the indicated genotypes was determined and plotted as bar graphs representing the mean ± SD. Data originate from analysis shown in Fig. 2. **(d)** Distance of *MyoD1* gene to the nuclear periphery was measured in wildtype

and knockout myoblasts and displayed in violin plots. Violin plots display data of Fig. 2 prior to normalization to nuclear height (absolute distances). Emerin knockout myoblasts show a subtle increase in the distance of the *MyoD1* gene to the nuclear periphery, which is not detected upon normalization of the distance by the nuclear height (see Fig. 2). H3K9me2-positive peripheral heterochromatin layer is marked as gray-shaded bar. n_{Wt}= 208, $n_{emerin\ KO}$= 333, $n_{LAP2\beta\ KO}$= 140; n_{Wt}= 722, $n_{LA/C\&LBR\ KO}$= 453, $n_{LA/C,\ LBR\&Emd\ KO}$= 300. $^{**}p_{Emd\ KO}$= 0.0065, other p values> 0.05 (two-sample, two-tailed KS test, Hochberg correction for multiple testing). **(e)** Total lamin B1 levels were tested by Western blot analysis in lamin A/C and LBR single and double knockout cells used for lamin B1 ChIP shown in Fig. 3e. Orange star marks unspecific band. **(f)** Wildtype and indicated knockout myoblasts were processed for Western blot analysis using antibodies to indicated antigens (MyoD1, total H3, H3K9me2, H3K9me3 and H3K27me3), revealing no changes in total protein levels in single and double knockout cells. **(g)** Levels of endogenous MyoD1 mRNA in lamin A/C-LBR double knockout myoblasts relative to wildtype cells, determined by quantitative qRT-PCR. Bar graph displays mean ± SEM from 5 biological replicates. t(8)= 0.82, p= 0.02 (Welch's t-test). KO: knockout; LA/C: lamin A/C. Images processed with Fiji. Immunofluorescence and immunoblot experiments shown as representative examples were repeated independently two times with similar results. Source data are provided as a Source Data file.

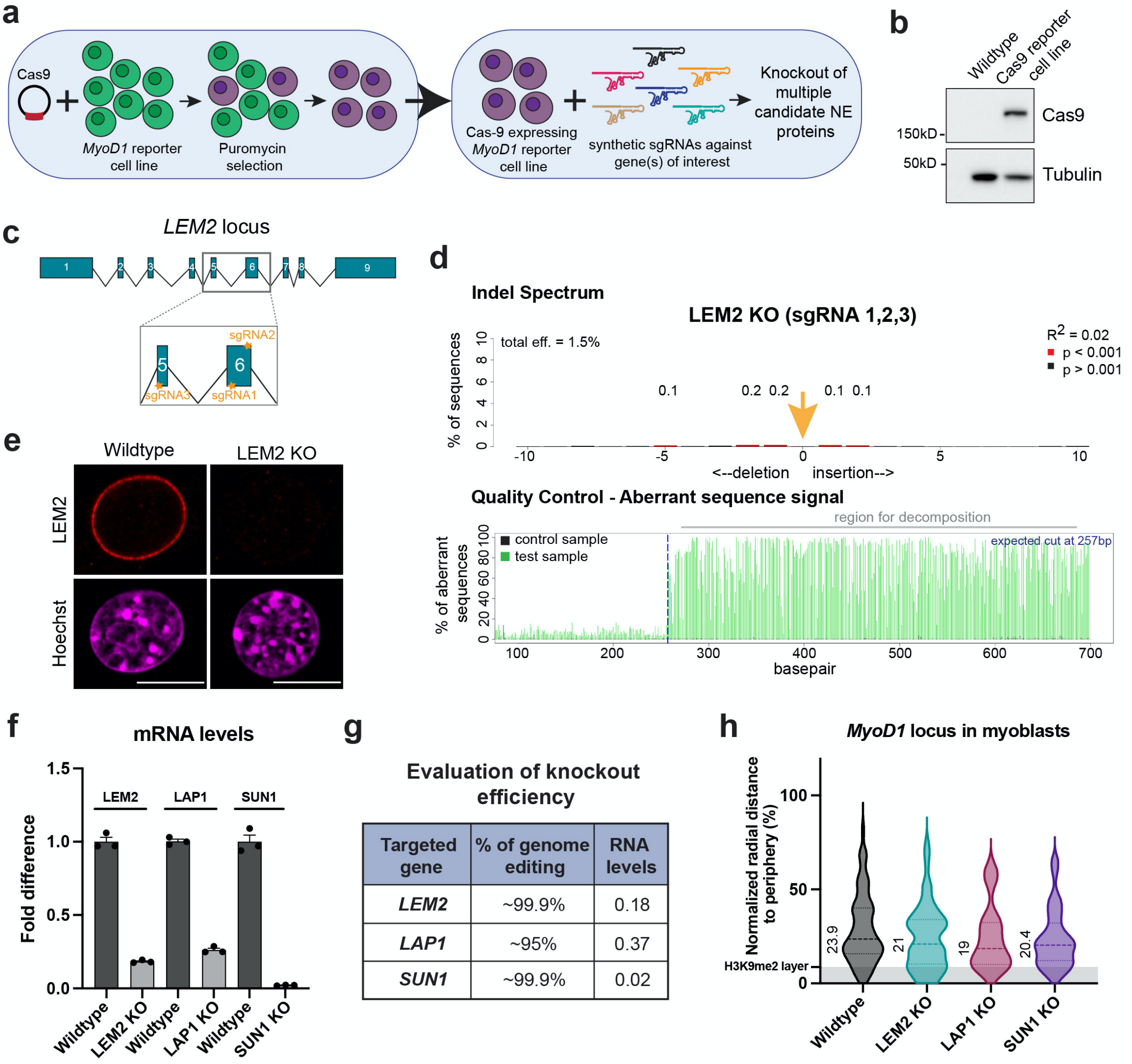

Supplementary Figure 4. CRISPR/Cas9-based strategy for multiple high efficiency protein knockout. (a) Schematic overview of knockout approach: *MyoD1* lacO-tagged C2C12 reporter cell lines were transduced with a plasmid expressing Cas9 and stably Cas9-expressing reporter lines were transfected with synthetic single-guide RNAs (sgRNAs) targeting a single or multiple genes of interest. **(b)** Western blot analysis confirming Cas9 expression in *MyoD1* lacO-tagged reporter cell line. Western blot analysis was repeated independently three times with similar results. **(c)** Schematic drawing of the mouse *LEM2* locus containing 9 exons (blue boxes) and introns (angled lines). Regions targeted by synthetic sgRNAs in exons 5 and 6 are indicated by orange stars (black box). **(d)** Targeting efficiency was determined using the TIDE software. The region spanning the editing site was amplified from genomic DNA of LEM2 knockout and wildtype cells by PCR. PCR products were sequenced and uploaded in TIDE. Aberrant sequence signal (lower panel), originating from distinct frame shifts following the repair of double strand breaks at the cut sites (dashed blue line), is decomposed and indels

of up to 50 bps are mapped. Targeting of genes with multiple sgRNAs often leads to longer deletions, which cannot be mapped by the TIDE software (upper panel). Position 0 (yellow arrow) shows percentage of unedited wildtype sequences, R^2 value demonstrates the goodness-of-fit, p values are calculated by two-tailed t-test of the variance-covariance matrix of the standard errors. **(e)** LEM2 protein levels were assessed by immunofluorescence analysis in wildtype and knockout cells. Scale bars, 10 μm. **(f)** LEM2, SUN1 and LAP1 transcript levels in knockout cells relative to wildtype cells were analyzed by RT-qPCR. Data represent mean values ± SEM from 3 technical replicates. **(g)** Table summarizes the efficiency of single knockouts of indicated proteins achieved using this approach. **(h)** Normalized distance of *MyoD1* to the nuclear periphery in C2C12 myoblasts of indicated genotypes. n_{Wt}= 129, $n_{LEM2\ KO}$= 98, $n_{LAP1\ KO}$= 19, $n_{SUN1\ KO}$= 45. p values> 0.05 (two-sample KS test, Hochberg correction for multiple testing). Numeric values displayed on violin plots represent median values. Images processed with Fiji. KO: knockout. Source data are provided as a Source Data file.

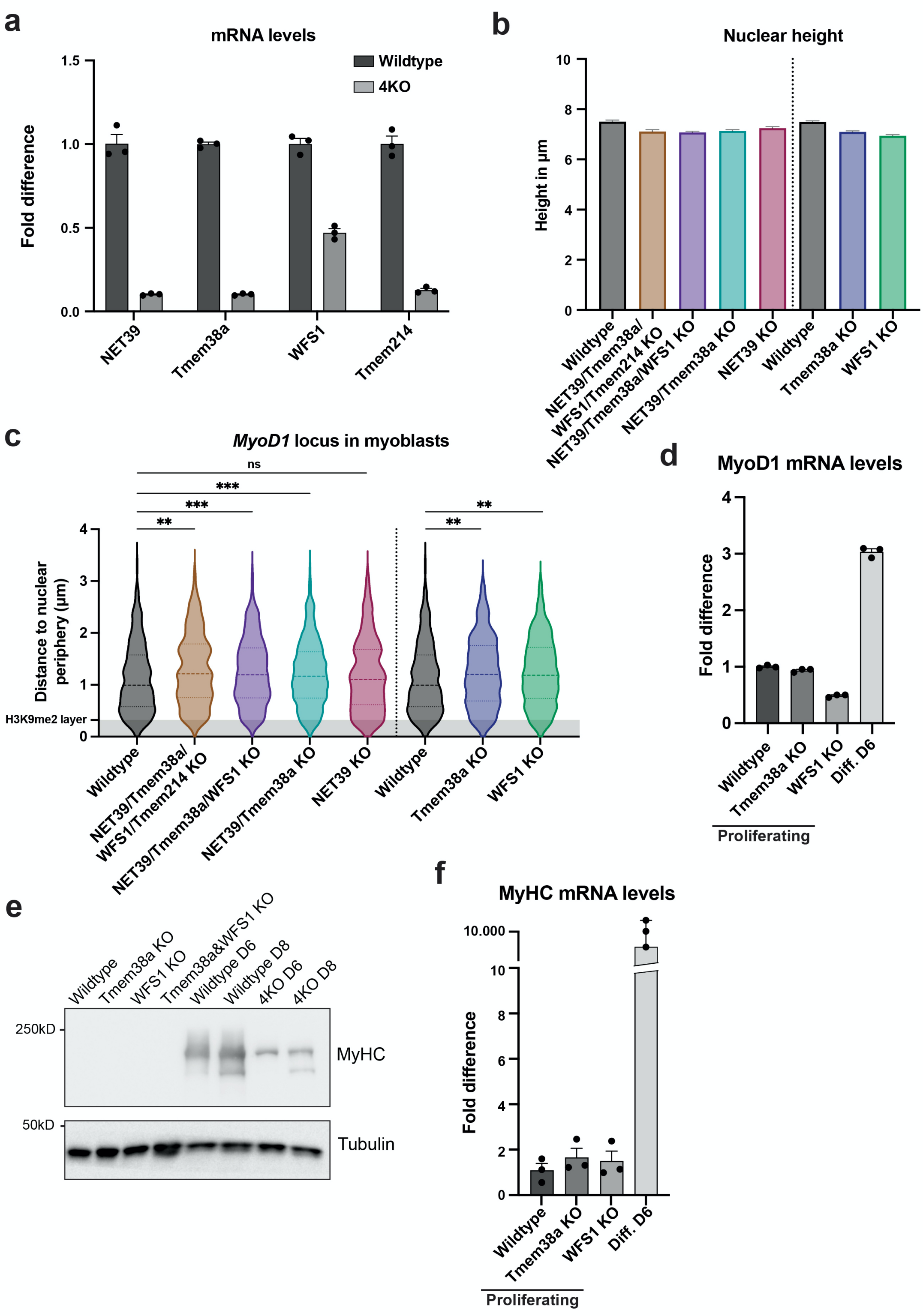

Supplementary Figure 5. *MyoD1* release from the nuclear periphery upon depletion of anchor proteins is not a result of premature induction of differentiation. (a) Endogenous mRNA levels of NET39, Tmem38a, WFS1 and Tmem214 in knockout cells with simultaneous depletion of all four genes (4KO) relative to wildtype control cells were analyzed by RT-qPCR. Data represents mean values ±

SEM of 3 technical replicates. **(b)** Nuclear height of cells with the indicated genotypes was determined and plotted as bar graphs representing the mean ± SD. Data originate from dataset presented in Fig. 4. **(c)** Distance of *MyoD1* to the nuclear periphery displayed as absolute values in violin plots. H3K9me2-positive heterochromatin layer at periphery is marked by a gray-shaded bar. n_{Wt}= 722, n_{4KO}= 454, n_{3KO}= 898, n_{2KO}= 685, $n_{NET39\ KO}$= 500; $n_{Tmem38a\ KO}$= 571, $n_{WFS1\ KO}$= 517. **p_{4KO}= 0.004, ***p_{3KO}= 1 x 10^{-4}, ***p_{2KO}= 1 x 10^{-4}, $p_{NET39\ KO}$= 0.5; **$p_{Tmem38a\ KO}$= 0.006, **$p_{WFS1\ KO}$= 0.006 (two-sample, two-tailed KS test, Hochberg correction for multiple testing). **(d)** Endogenous mRNA levels of MyoD1 in Tmem38a and WFS1 knockout proliferating C2C12 myoblasts and in wildtype differentiating myotubes relative to wildtype proliferating myoblasts were determined by RT-qPCR. Data represents mean values ± SEM from 3 technical replicates. Diff. D6: differentiation day 6. **(e, f)** Premature differentiation of Tmem38a and WFS1 KO cells was assessed by determining the level of the differentiation marker myosin heavy chain (MyHC) in proliferating knockout versus wildtype cells by **(e)** Western blot analysis and **(f)** RT-qPCR. D6: day 6; D8: day 8; Diff. D6: differentiation day 6. Data represents mean values ± SEM from 3 technical replicates. KO: knockout. Western blot experiment shown as representative example was repeated independently two times with similar results. Source data are provided as a Source Data file.

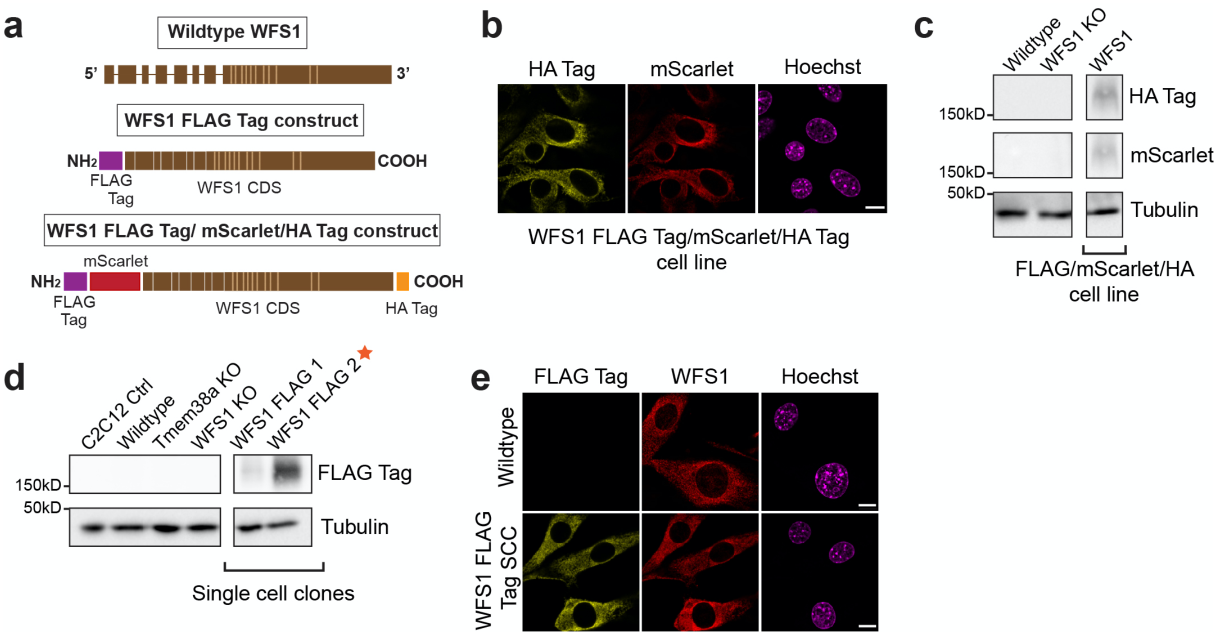

Supplementary Figure 6. Generation and characterization of WFS1-depleted myoblasts expressing ectopic tagged WFS1. (a) Schematic representation of the mouse *WFS1* gene locus including 8 exons (brown boxes) and introns (lines) (top panel), and of ectopic WFS1 protein containing an N-terminal FLAG-tag (middle panel), or N-terminal FLAG- and mScarlet- tags, and a C-terminal HA-tag (bottom panel). Light brown lines designate predicted transmembrane domains. NH_2: N-terminus; COOH: C-terminus; CDS: coding sequence. **(b, c)** Expression of the FLAG-, mScarlet- and HA-tagged WFS1 protein (shown in a) was analyzed by **(b)** immunofluorescence, and **(c)** immunoblot analysis using indicated antibodies. **(d)** Ectopic expression of FLAG-tagged WFS1 in single cell clones (SCC) was verified by Western blot analysis in indicated cell lines using an antibody to the FLAG tag. Orange star marks cell clone used for further experiments. **(e)** Expression and correct localization of the FLAG-WFS1 protein was assessed by immunostaining using anti-FLAG Tag and anti-WFS1 antibodies. Scale bars, 10 µm. Images processed with Fiji. KO: knockout; Ctrl: control. Immunofluorescence and immunoblot experiments shown as representative examples were repeated independently two times with similar results. Source data are provided as a Source Data file.

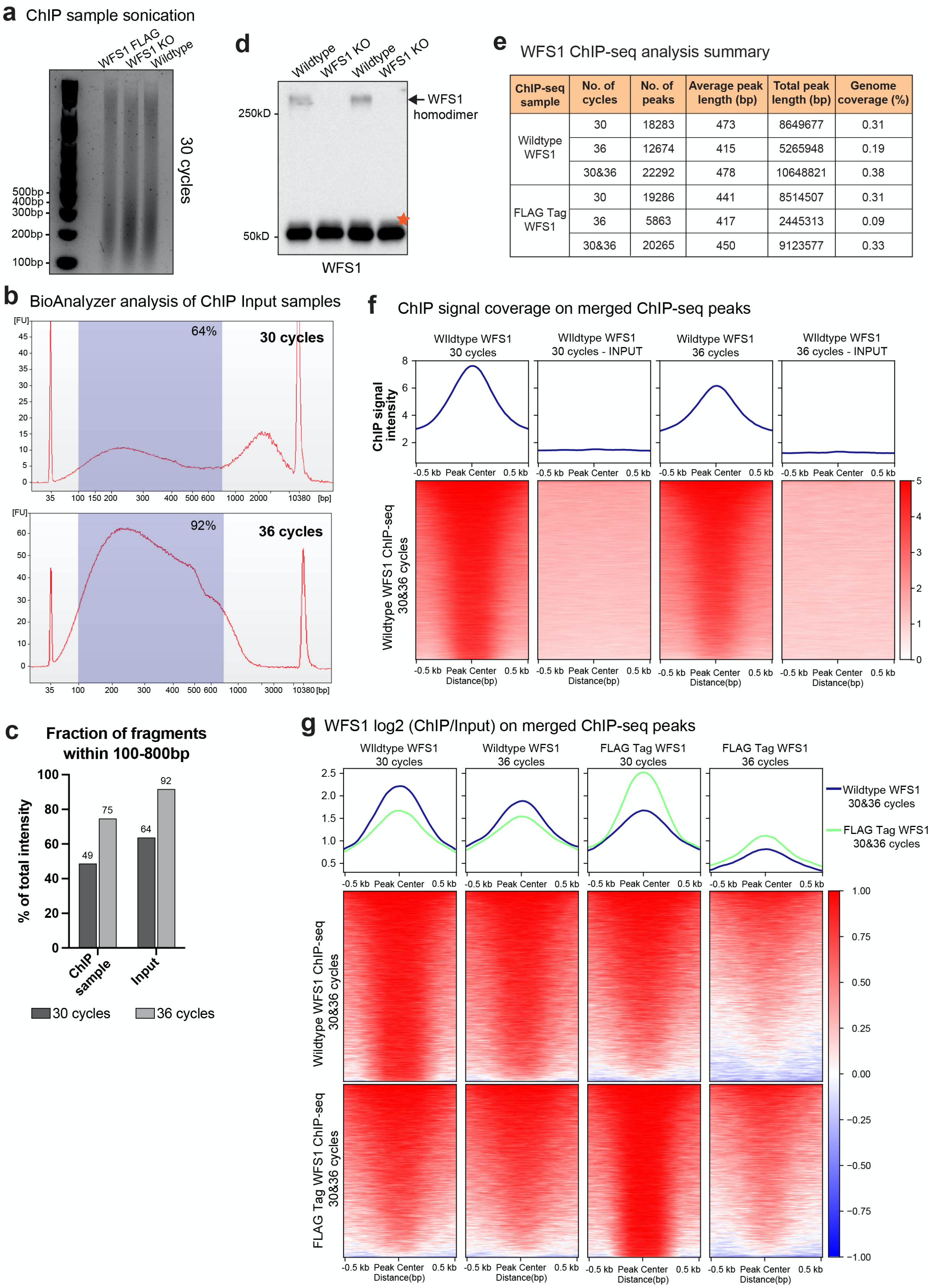

ChIP-seq sample	No. of cycles	No. of peaks	Average peak length (bp)	Total peak length (bp)	Genome coverage (%)
Wildtype WFS1	30	18283	473	8649677	0.31
	36	12674	415	5265948	0.19
	30&36	22292	478	10648821	0.38
FLAG Tag WFS1	30	19286	441	8514507	0.31
	36	5863	417	2445313	0.09
	30&36	20265	450	9123577	0.33

Supplementary Figure 7. Characterization of WFS1 ChIP-seq genome-wide analysis. (a) Fragment size assessment of sonicated chromatin isolated from wildtype, WFS1 knockout and WFS1 FLAG cell

lines was performed using a 1.8% agarose gel. **(b)** Representative Bioanalyzer intensity profiles of wildtype input ChIP samples following 30 and 36 sonication cycles, as indicated. Purple boxes indicate selected fragment size range used for library preparation (100-800 bp). **(c)** Bar graph displays quantification of chromatin fragment content within 100-800 bp range in ChIP and input samples following 30 and 36 sonication cycles. **(d)** Western blot analysis confirming specific WFS1 detection in the chromatin fraction used for ChIP analysis of wildtype and WFS1 knockout cells. Cells were processed using the WFS1 ChIP protocol (see Methods, two-step crosslinking, 30 sonication cycles, immunoprecipitation protocol). Samples for protein identification were obtained following WFS1 immunoprecipitation and prior to protein degradation, orange asterisk denotes unspecific band. **(e)** Table summarizes genome coverage and key parameters of MACS peaks for WFS1 ChIP-seq analysis of endogenous and ectopic FLAG-tagged WFS1 expressed in WFS1 knockout cells. (f) Average binding profiles and heat maps showing enrichment of WFS1 signal (RPKM) in individual ChIP experiments using 30 and 36 cycles of sonication on merged peaks (30 plus 36 cycles) ± 0.5 kb from center and on same regions in respective input samples. Heat maps are ranked according to WFS1 enrichment in descending order. (g) Average binding profiles and heat maps showing enrichment of WFS1 and FLAG Tag WFS1 signals (log2[ChIP/input]) in individual ChIP experiments using 30 and 36 cycles of sonication on merged WFS1 peaks (30 plus 36 cycles) ± 0.5 kb from center. Heat maps are ranked according to WFS1 enrichment in descending order. KO: knockout. Source data are provided as a Source Data file.

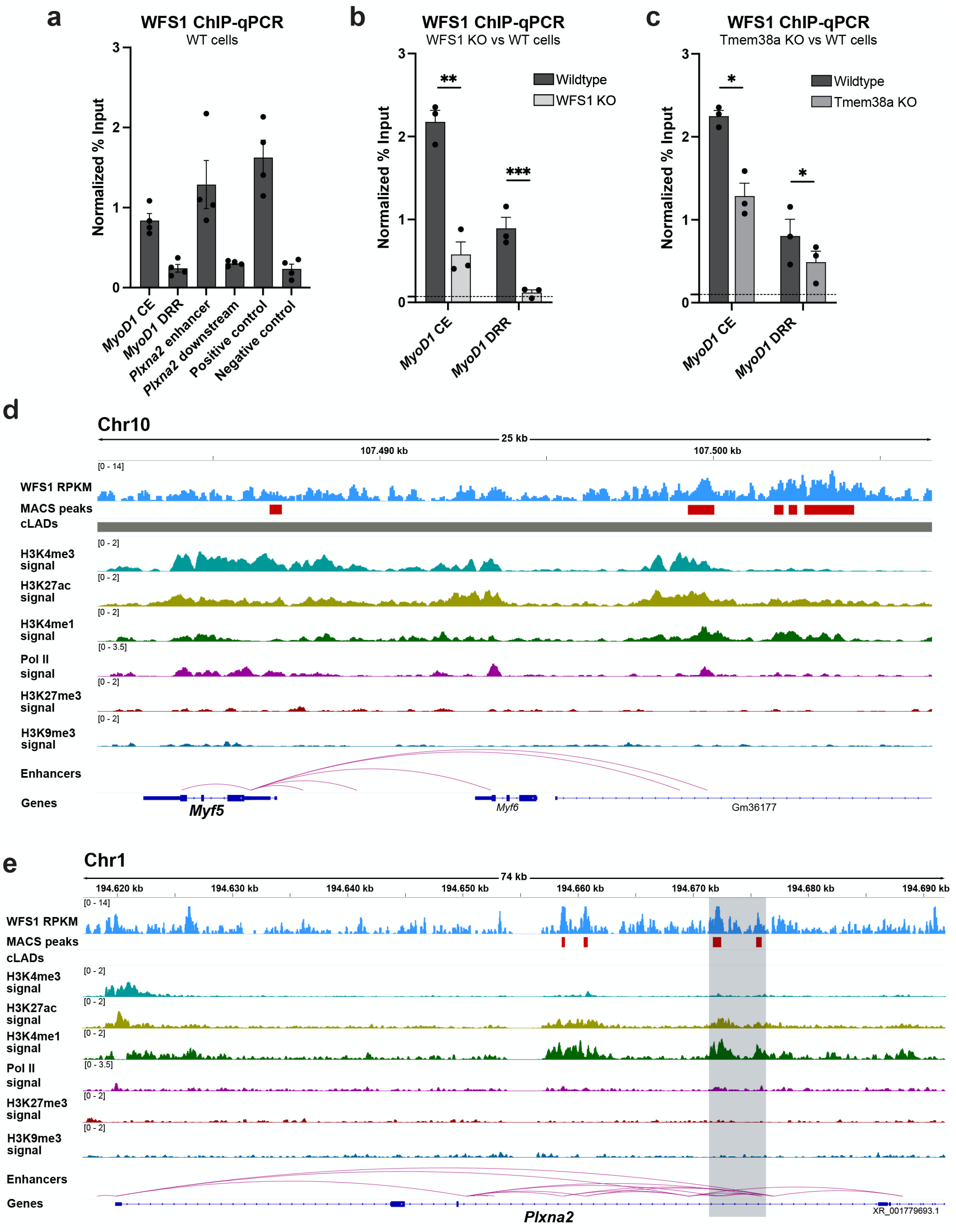

Supplementary Figure 8. WFS1 ChIP signal is specifically enriched in enhancer regions of genes linked to muscle differentiation. (a) WFS1 ChIP-seq signal specificity was assessed by chromatin immunoprecipitation (ChIP) coupled with qPCR analysis of predicted enhancer regions (*MyoD1* CE, *Plxna2* enhancer, positive control) displaying high enrichment of WFS1 signal in ChIP-sequencing

(ChIP-seq) analysis and regions (*MyoD1* DRR, *Plxna2* downstream, negative control), where WFS1 signal is depleted in ChIP-seq analysis of wildtype (WT) myoblasts. Bar graph displays mean ± SEM from 4 biological replicates. Positive control coordinates chr15: 62328465 – 62328694; Negative control coordinates chr10: 127033080 – 127033322. **(b, c)** Abundance of WFS1 signal on the *MyoD1* CE and *MyoD1* DRR regions in wildtype (WT) versus (b) WFS1 knockout (KO) and (c) Tmem38a knockout (KO) proliferating C2C12 myoblasts was assessed by ChIP-qPCR analysis. Bar graph displays mean ± SEM from 3 biological replicates. $t_b(3)_{MyoD1\ CE} = 3.56$, $t_b(3)_{MyoD1\ DRR} = 5.43$, $**p = 0.0075$, $***p = 0.0006$, $t_c(3)_{MyoD1\ CE} = 3.98$, $t_c(3)_{MyoD1\ DRR} = 3.66$, $*p_{MyoD1\ CE} = 0.03$, $*p_{MyoD1\ DRR} = 0.04$ (two-way ANOVA test). Dashed horizontal line indicates background ChIP signal obtained with IgG control. **(d, e)** IGV tracks of mouse genomic regions (mm10) containing the genes **(d)** *Myf5* and **(e)** *Plxna2* suggested to associate with the nuclear periphery in C2C12 cells and their predicted regulatory elements. Included are WFS1 ChIP tracks (RPKM, reads per kilobase per million) following sonication for 30 cycles, WFS1 peak regions identified by MACS, constitutive LAD regions (cLADs), ChIP-seq signal tracks of histone modification H3K4me3, H3K27ac, H3K4me1, H3K27me3 and H3K9me3, RNA polymerase II (Pol II) ChIP-seq signal track, enhancer predictions and gene annotations from NCBI reference sequence database gene track. Grey box outlines *Plxna2* enhancer region tested by ChIP RT-qPCR in panel a. Source data are provided as a Source Data file.

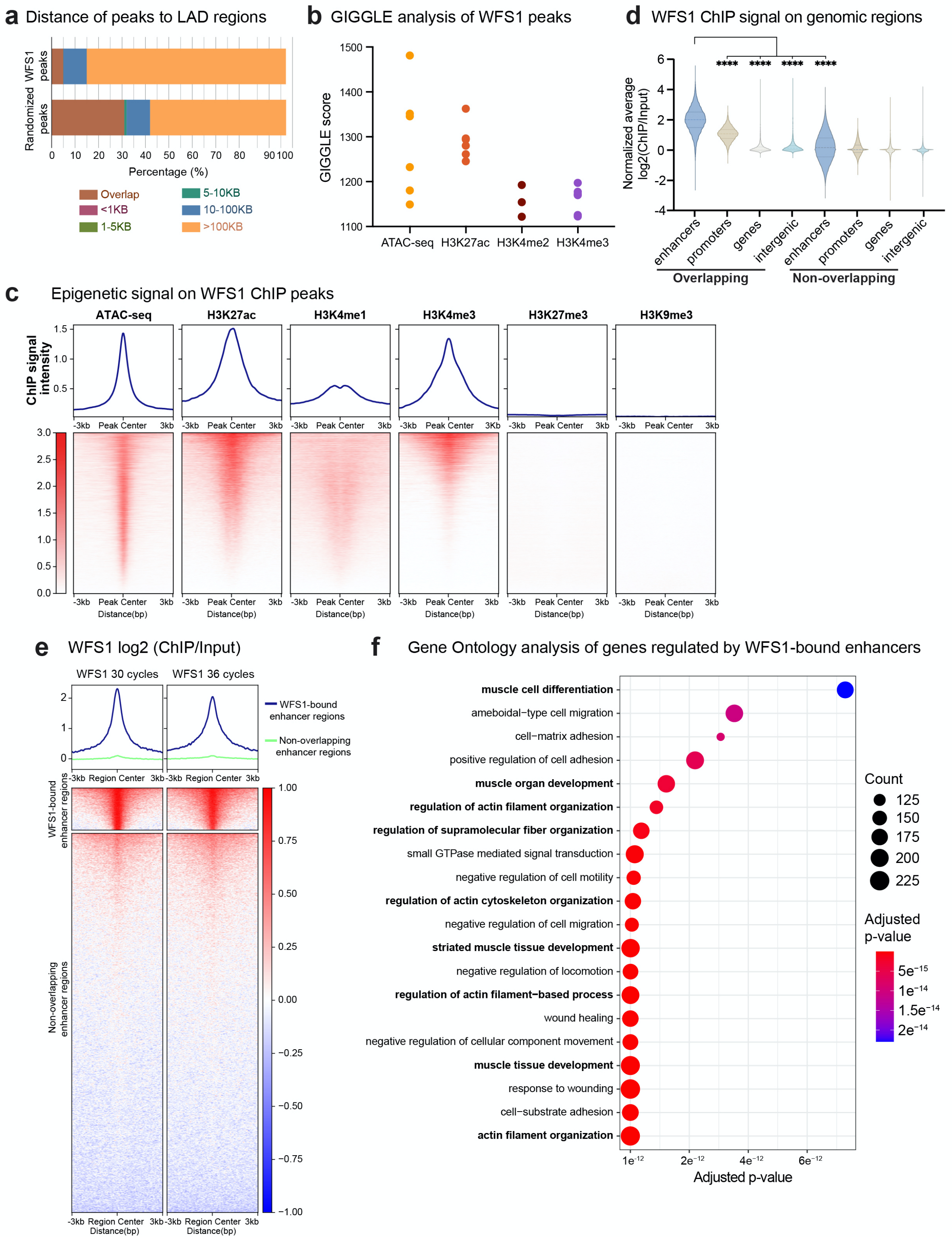

Supplementary Figure 9. WFS1 ChIP-seq genome-wide analysis reveals high correlation with active marks and low binding to heterochromatic LAD regions. (a) Graph indicates distances of WFS1 MACS peaks or randomized peaks to the closest LAD region. Data are displayed as percentage of total peaks within a range of distances (bins specified on the right). **(b)** Dot blot graph shows GIGGLE

scores representing the highest similarities of WFS1 ChIP-sequencing (ChIP-seq) peaks to all histone mark datasets available on the Cistrome database. Similarities are revealed through the GIGGLE score, a combination of the estimated significance and enrichment between the pair-wise comparisons. **(c)** Average binding profiles and heat maps showing ATAC-seq, H3K27ac, H3K4me1, H3K4me3, H3K27me3 and H3K9me3 ChIP-seq signal intensity on WFS1 MACS peaks ± 3 kb from peak center. Heat maps are ranked in descending order of ChIP-seq signal intensity. **(d)** Violin plots represent the normalized average signal intensity (log2[ChIP/Input]) in different genomic regions within (overlapping) and outside (non-overlapping) of WFS1 peaks. ****p values< 1×10^{-15} (Kruskal-Wallis test, Dunn's correction for multiple testing); overlapping n: $n_{enhancers} = 17{,}787$, $n_{promoters} = 4{,}288$, $n_{genes} = 5{,}907$, $n_{intergenic} = 5{,}481$; non-overlapping n: $n_{enhancers} = 311{,}775$, $n_{promoters} = 51{,}036$, $n_{genes} = 32{,}268$, $n_{intergenic} = 32{,}673$; effect size was calculated according to Cohen's *d*, $d_{enh./prom.}= 1.13$, $d_{enh./genes}= 2.21$, $d_{enh./inter.}= 2.14$, $d_{enh./non\text{-}ov.enh.}= 1.99$. **(e)** Average binding profile and heat map displaying enrichment of WFS1 (log2[ChIP/input]) on predicted WFS1-bound enhancer regions ± 3kb from center (top) and on predicted enhancers outside of WFS1 peaks ± 3kb from center (bottom). Heat maps are ranked according to WFS1 enrichment in descending order. **(f)** Gene ontology (GO) enrichment analysis of all genes associated with predicted WFS1-bound enhancers. Chart shows the 20 top GO terms for biological process ranked by fold enrichment (adjusted p value). Processes involved in muscle differentiation and actin organization are highlighted in bold.

SUPPLEMENTARY METHODS

Generation of repair template vector

The repair template construct used for CRISPR-mediated homology-directed repair was assembled from four fragments; the left and right homology arms, the 64xLacO repeats, as well as the pUC18 backbone vector. The left and right homology arms were amplified by PCR using mouse genomic DNA as a template and the primer pairs listed in Supplementary Table 1 (for primer sequences see Supplementary Table 4).

Supplementary Table 1. Generation of left and right homology arms.

Fragment	F primer	R primer
MyoD1 LHA	*MyoD1* LHA-F	*MyoD1* LHA-R
MyoD1 RHA	*MyoD1* RHA-F	*MyoD1* RHA-R
Pax7 LHA	*Pax7* LHA-F	*Pax7* LHA-R
Pax7 RHA	*Pax7* RHA-F	*Pax7* RHA-R

A plasmid containing 64xLacO repeats[1] (a generous gift by Andrew S. Belmont) was isolated from *E.coli* using the Monarch® Plasmid Miniprep Kit #T1110 and digested with BamHI and SalI (New England Biolabs #R3136S and #R3138S). The vector pUC18[2] was digested with HindIII and SmaI (New England Biolabs #R3104S and #R0141S). All four fragments were assembled using NEBuilder® HiFi DNA Assembly mix (New England Biolabs #E2621S) according to manufacturer's instructions. The final construct was sequenced for verification and amplified in high-efficiency NEB5-alpha competent *E. coli* from New England Biolabs #C2987H.

Genotyping PCR and long range (LR) PCR for modified *MyoD1*/*Pax7*-LacO knock-in allele

For the identification of single cell clones with modified *MyoD1* and *Pax7* alleles, genomic DNA was isolated with QuickExtract DNA extraction solution (Epicentre/Lucigen #QE09050) according to manufacturer's instructions and the presence of wildtype and knock-in alleles was determined by PCR (see Supplementary Table 2) using the GoTaq green master mix (Promega #M7122). Clones carrying at least one knock-in allele were further analyzed by LR-PCR using the Q5 DNA polymerase (New

England Biolabs) to verify correct integration of the construct (see Supplementary Table 2, see also Supplementary Fig. 1a-d). For primer sequences see Supplementary Table 4.

Supplementary Table 2. Genotyping and long range (LR) PCRs.

PCR	Target	Forward primer	Reverse primer
WT	*MyoD1*	*MyoD1*-F	*MyoD1* WT-R
	Pax7	*Pax7*-F	*Pax7* WT-R
Knock-in	*MyoD1*	*MyoD1*-F	KI-R
	Pax7	*Pax7*-F	KI-R
LR 5’ PCR	*MyoD1*	*MyoD1* 5' LR-F	KI-R
	Pax7	*Pax7* 5' LR-F	KI-R
LR 3’ PCR	*MyoD1*	3' LR-F	*MyoD1* 3' LR-R
	Pax7	3' LR-F	*Pax7* 3' LR-R

Vectors for cell lines with tagged WFS1

Vectors expressing differently tagged versions of WFS1 were assembled using NEBuilder® HiFi DNA Assembly mix (New England Biolabs #E2621S). Plasmid pcDNA3.1 (Addgene #52535) was digested with BglII and EcoRI for assembly 1, and BglII and BamHI for assembly 2 (New England Biolabs #R0143S, #R3101S and #R3136S respectively). Different fragments were generated by PCR using the primer pairs (for primer sequences see Supplementary Table 4) and templates listed in Supplementary Table 3.

Supplementary Table 3. PCRs for vector assembly

Assembly 1 Fragment	Forward primer	Reverse primer	Template
PGK promoter	PGK promoter-F	PGK promoter-R	Kindly gifted from Nicolaus Beer
FLAG Tag	FLAG Tag-F	FLAG Tag-R	Kindly gifted from Daria Filipczak
WFS1 coding sequence A	WFS1 CDS A-F	WFS1 CDS A-R	Twist Bioscience sequence A
WFS1 coding sequence B	WFS1 CDS B-F	WFS1 CDS B-R	Twist Bioscience sequence B
Assembly 2 Fragment	Forward primer	Reverse primer	Template
PGK promoter	PGK promoter-F	PGK promoter-R	Kindly gifted from Nicolaus Beer

FLAG Tag	FLAG Tag-F	FLAG Tag-R2	Kindly gifted from Daria Filipczak
WFS1 coding sequence	WFS1 CDS HA-F	WFS1 CDS HA-R	WFS1-FLAG Tag plasmid
mScarlet	mScarlet-F	mScarlet-R	Addgene #85042

The HA Tag for assembly 2 was added as a flanking sequence to the primers used for PCR amplification. Finally, the two vectors of interest (see also Supplementary Fig. 7a) were assembled using the fragments shown in the tables above using NEBuilder HiFi DNA assembly mix (New England Biolabs #E2621S) following the manufacturer's instructions.

Supplementary Table 4. Primers used for cell line generation

Primer	Sequence (5' - 3')
MyoD1 LHA-F	AACGACGGCCAGTGCCAAGCTTCAGCATAGTGGAGCGCATCTCC
MyoD1 LHA-R	AATTCGGCGCCTCTAGAGTCGACGTCTACACAGTCTGATGGAAGCACC
MyoD1 RHA-F	ATGTGGAATTCCTCGAGGGATCCGGCAGGGGGAGGCCATTGGT
MyoD1 RHA-R	CGAATTCGAGCTCGGTACCCGGGGCTGTATAGCTGGTAGGTAGGTCTCTGGA
Pax7 LHA-F	AAACGACGGCCAGTGCCAAGCTTGGCAAACTGACTCTCTGGTAGC
Pax7 LHA-R	AATTCGGCGCCTCTAGAGTCGACTGTGGTTCCCAGACACCCTGGT
Pax7 RHA-F	ATGTGGAATTCCTCGAGGGATCCCGATGGGTTGGTGTGGGAGTG
Pax7 RHA-R	CGAATTCGAGCTCGGTACCCGGGAGGGCTCAGAGCTGTATCCG
MyoD1-F	TGGCAGAATGAGTCTGTCTAGGA
MyoD1 WT-R	AATGGCCTCCCCCTGCCGTCTA
Pax7-F	GCAGGATGCTTCTCTTGGGTA
Pax7 WT-R	TCCCACACCAACCCATCGTGTG
KI-R	GAATTCGGCGCCTCTAGAGTCGAC
MyoD1 5' LR-F	ACAGAGTCCAGGCCAGGGAAGAGTG
Pax7 5' LR-F	GACACCAGGGACACTTGTCTACATG
3' LR-F	CATGTGGAATTCCTCGAGGGATCC
MyoD1 3' LR-R	GCAGACTGGGACATGGTGTGTTCA
Pax7 3' LR-R	CACAGCAACATGCTTCCTGCCAT
PGK promoter-F	GACGTCGACGGATCGGGAGATCTGGGTAGGGGAGGCGCTTTTCC

PGK promoter-R	CATGGTGGCACCGGCGCTGCAGGTCGAAAG
FLAG Tag-F	AGCGCCGGTGCCACCATGGACTACAAAGACCATGAC
FLAG Tag-R	TGCCTGAGTTTCTAGAGGGCCCCTTGTCATCG
WFS1 CDS A-F	CCCTCTAGAAACTCAGGCACCCCACCTCC
WFS1 CDS A-R	GCTGACGTTGAGGATGATGAAGTGGCCAACGGGCACAG TGAAGAAAGTC
WFS1 CDS B-F	CCGTTGGCCACTTCATCATCCTCAACGTCAGCCTC
WFS1 CDS B-R	CACTAGTCCAGTGTGGTGGAATTCTCAGGCGGCAGACA GGAATGG
FLAG Tag-R2	CGCCCTTGCTCACAGAACCACCTCCGCCCTTGTCATCGT CATCCTTGTAATC
WFS1 CDS HA-F	GAGCTGTACAAGGGGCCCTCTAGAAACTCAGGCACCC
WFS1 CDS HA-R	AAACTTAAGCTTGGTACCGAGCTCGGATCCTCAAGCGT AATCTGGAACATCGTATGGGTAAGAACCACCTCCGCCG GCGGCAGACAGGAATGGGAAGAAG
mScarlet-F	GACAAGGGCGGAGGTGGTTCTGTGAGCAAGGGCGAGG CAGTGAT
mScarlet-R	GAGTTTCTAGAGGGCCCCTTGTACAGCTCGTCCATGCCG CC

Supplementary Table 5. Primers used for TIDE analyses

TIDE primers	Sequence (5' – 3')
Emd TIDE-F	AAAGGAAGTCTCAACCGCTGCC
Emd TIDE-R	GGTGAGGGAGGTCTGGGGTTT
LBR TIDE-F	CCATGTGGGTGCTGGGTCCT
LBR TIDE-R	GTTCTGCGCACACAGAGCAC
LAP2β TIDE-F	TCCAGGGCTAGGTCAGTTCCC
LAP2β TIDE-R	AGTCAACCTATTGGCCACCTGC
LMNA TIDE-F	CCTGTAGAGGAGGGCCTATTAGA
LMNA TIDE-R	ACCCCCTCCCTTCTATGTCC
LEM2-1 TIDE-F	CACCTCCTGAGTGGAGCCGGT
LEM2-1 TIDE-R	GCCTCTTGCCACCTGGCGTT
LEM2-2 TIDE-F	AGCCGCTCAGGGCAGCTGTTT
LEM2-2 TIDE-R	CTGGGCTTCCCTCCCACGGT
NET39-F	AGGTCACCATGCCAGCTTCCCA
NET39-R	TGACCAAGACATCACACTAGGCCAC
SUN1-1-F	TTGCAGGGACTGGAAACGGAG
SUN1-1-R	ATCCAGAGGATGGCCAATGCAG
SUN1-2-F	TGACAGCGTGGCACCATCAT
SUN1-2-R	TCCAAATACTTCAGGGCTGGGGA
SUN1-3-F	GGTTTTACTTGGGGGTGAAGGGG
SUN1-3-R	AGCATGCACCTCCCAAAGACAG

LAP1-1-F	ATGGTCGTAGGCACCATAGCAC
LAP1-1-R	GGTCTACTGTGACAATTTCCAGGTT
LAP1-2-F	GCTAGAGGTTCCTAAAACTGAGCA
LAP1-2-R	AGCCTCAGTGTATGGTTTTAGACA
Tmem38a-F	GCCTTGAACTCACAGATCTGCCT
Tmem38a-R	GGCACACTGGTGGTTGAGCTT
WFS1-1-F	GCCTTGGTCCACCAACGGAAA
WFS1-1-R	TCGCCTTCTTCATCCCCCTGG
WFS1-2-F	GCGCAGGACGGACATACTCAC
WFS1-2-R	ACGGTGCCAGTCAGAACTCC
WFS1-3-F	CGGCAACTTCAGCTGGTACG
WFS1-3-R	GGTACCCCAAAAGGCAAGGTGA
Tmem214-1-F	CGTGTTCACTGGGAACCCCTC
Tmem214-1-R	AGTACGCGTGGGGAATGCTA
Tmem214-2-F	CTGAGTTCCTTCCCCAGGGTTC
Tmem214-2-R	GACTGGGCTGTACCACAGCAA

Supplementary Table 6. sgRNAs used to generate knockout cell lines

Oligo sgRNAs	Sequence (5'-3')
Emd sgRNA 1-F	CACCGAGAAGGAGACTCTTACCCC
Emd sgRNA 1-R	AAACGGGGTAAGAGTCTCCTTCTC
Emd sgRNA 2-F	CACCGTTGTACTGGCGTAGCACTG
Emd sgRNA 2-R	AAACCAGTGCTACGCCAGTACAAC
LAP2β sgRNA 1-F	CACCGAGGGTCGAGAAGAACTCCA
LAP2β sgRNA 1-R	AAACTGGAGTTCTTCTCGACCCTC
LAP2β sgRNA 2-F	CACCGCCTTTATAGTTTCAGCTAT
LAP2β sgRNA 2-R	AAACATAGCTGAAACTATAAAGGC
LMNA sgRNA 1-F	CACCGGGGGACTTGTTGGCTGCGC
LMNA sgRNA 1-R	AAACGCGCAGCCAACAAGTCCCCC
LMNA sgRNA 2-F	CACCGTGATCCGAGTGGGCGACAG
LMNA sgRNA 2-R	AAACCTGTCGCCCACTCGGATCAC
Dharmacon sgRNAs	Sequence (5'-3')
Plpp7 (NET39)-1	TGCAGATGTCAATAGCCAGC
Plpp7 (NET39)-2	GGGCACACTCACCTAGCAGC
Plpp7 (NET39)-3	GATGCCAATGAGCTTGACCA
Lemd2-1	CTGTAGTCGCCAGCTCAGAT
Lemd2-2	CCAGAAGAAGATGAGCACGT
Lemd2-3	CCTGAGCAGCAACAAGGACG
Sun1-1	ACATGGCTGGAGTGCTCTAC
Sun1-2	AGTTGGAGAGACGCACTTCA
Sun1-3	AGTGTTCTCCGGCACACACT

Tor1aip1(LAP1)-1	GTCTGCATAAGTTGCTTATA
Tor1aip1(LAP1)-2	TTACAGTGACATCACTATCA
Tor1aip1(LAP1)-3	CCATTAAGAAGCCCAAGACT
Tmem38A-1	GTTGCTGAAGTAGTCAATGA
Tmem38A-2	GCACACAGCCAGGACGCCAC
Tmem38A-3	TTGCTGAAGTAGTCAATGAT
Tmem214-1	TTGTGAGCCGAGAGCTACGT
Tmem214-2	GCACACACTTGCTGGCCAAC
Tmem214-3	CACGTAGCTCTCGGCTCACA
WFS1-1	GTCATCCTGCTCCAGTCTAC
WFS1-2	ATGACGGCCAGCTCCGAGCA
WFS1-3	TGTTGGACAGGTCAACGAGC
WFS1-4	GTGGAGGAGGTGGGCCAGAG

Supplementary Table 7. Primary antibodies used for immunoblotting and immunofluorescence experiments

Antigen	Host	WB Dilution	IF Dilution	Source
Lamin A/C	Mouse	1:1000	1:100	Santa Cruz (clone E1) sc-376248
LAP2alpha	Rabbit	N/A	1:1000	245-2[3]
Emerin	Rabbit	1:1000	1:100	Atlas Antibodies (HPA000609) Lot #A114889
LBR	Guinea pig	1:100	1:800	Gifted by Harald Herrmann
HA Tag	Mouse	1:1000	1:100	Sigma Aldrich (clone HA-7) H9658
FLAG Tag	Mouse	1:2000	1:100	Sigma Aldrich (clone M2) F1804
γ-tubulin	Mouse	1:5000	N/A	Sigma Aldrich #T5326 (clone GTU-88)
WFS1	Rabbit	1:400	1:100	Proteintech (26995-1-AP)
Cas9	Mouse	1:300	1:50	Generated by Stefan Schüchner, Max Perutz Labs, clone 7A9
RFP	Rat	N/A	1:1000	Proteintech (clone 5F8)
RFP	Mouse	1:2000	N/A	Proteintech (clone 6G6)
LEMD2	Mouse	1:2500	1:100	Atlas Antibodies (HPA017340) Lot #A60701
LAP2beta	Rabbit	1:10	Undiluted	Generated by C. Harris (mAK17, aa313-330)
H3K9me3	Rabbit	1:1000	1:800	Abcam (ab8898) Lot #GR3365652-1
H3	Rabbit	1:1000	N/A	Abcam (ab1791) Lot #GR153323-6
H3K9me2	Rabbit	1:1000	1:800	Active Motif (39420) Lot #34718002
H3K27me3	Rabbit	1:1000	N/A	Merck-Millipore (07-449) Lot #2275589
MyoD1	Mouse	1:600	N/A	Santa Cruz (clone G-1) sc-77460

Myogenin	Mouse	1:50	1:10	Santa Cruz (clone F5D) sc-12732
MyHC	Mouse	1:50	1:10	Invitrogen (clone MF20) 14-6503-82
Lamin B1	Rabbit	1:500	1:50	Proteintech (12987-1-AP) Lot #44-161-2324577

Supplementary Table 8. Primers used for ChIP-qPCR and qRT-PCR

ChIP-qPCR primers	Sequence (5’ – 3’)
LAD1-F	ACGGCTCCAATCTAAGGCCC
LAD1-R	TCCATCCGGCACAGTGTTCA
LAD2-F	ACTGCTGCAAGTAGCACCCA
LAD2-R	ATGGCCGCTGATCTCTCGTC
LAD3-F	GCAAGGCACTTTGCGCACTA
LAD3-R	GCAGCTTCATTTCGCTCCTCC
MyoD1 promoter-F	GGACAGCGCTGGGGTTCTAA
MyoD1 promoter-R	AGCCAAGGACAAAGTGGGCA
MyoD1 CE-F	GCCAAGTATCCTCCTCCAGC
MyoD1 CE-R	CTGTGTTGTGAGTCACGGGT
MyoD1 DRR-F	GGCCTAGCCAGACCAACATT
MyoD1 DRR-R	TCAGCTCCCTTGGCTAGTCT
Positive enhancer-F	TGGAATGCCACCGAAAAGGA
Positive enhancer-R	AAAGCCCCGAGGATTGTGAG
Negative enhancer-F	CGGGAAGGTAGGTTCCAGGT
Negative enhancer-R	GATAACGTCCACGCCAATGTG
Plxna2 enhancer-F	TGACCATGTGCCTGTGAGTC
Plxna2 enhancer-R	AGGTAGGGTGTGTGGGAACT
Plxna2 downstream-F	ACCTGCCACCATGGAATGAG
Plxna2 downstream-R	GCAGGTATCCAGGGTTGCTT
qRT-PCR primers	Sequence (5’ – 3’)
MyoD1-F	GCAGGCTCTGCTGCGCGACC
MyoD1-R	TGCAGTCGATCTCTCAAAGCACC
LEM2-F	TGGAGGCGCAGGAGTACATAG
LEM2-R	TCTTCCCCTTTCAGCCAGATGC
LAP1-F	GTGAGGACCGCGAAGAGGAT
LAP1-R	TCATCACTGAAGCCTCTGGTGT
SUN1-F	CCATGAAGTGCGTCTCTCCAAC
SUN1-R	GCTCTTCATCCCTGCTGCCT
NET39-F	AACCTGCTGCTAGCCCTGCT
NET39-R	GGCCGCGGCGCTTGATGA
Tmem38a-F	TGAAGTATGAGCCAGGAGCTGT
Tmem38a-R	GCAGGTCGGCCAGGATGTAA
WFS1-ex2-F	CTGGTCCCATGAAGGCAGATG
WFS1-ex2-R	GGTAGTGTTTGCCCACCTCAG

WFS1-ex4-F	CCTAGCTGACCGGAAAGGCAT
WFS1-ex4-R	TCCAGTACATGACCAGGGCAG
WFS1-ex7-F	CATTCCCACCAACCTGTTCCT
WFS1-ex7-F	AAGGGTACTTCACCACCTTCTG
Tmem214-F	TGAGCCAGTATCCACATGATTATCC
Tmem214-R	AGACCCTGCAGCTTTGGTCAG
MyHC-F	ACAAGCTGCGGGTGAAGAGC
MyHC-R	CAGGACAGTGACAAAGAACG
GAPDH-F	TGTGAACGGATTTGGCCGTA
GAPDH-R	ACTGTGCCGTTGAATTTGCC

SUPPLEMENTARY REFERENCES

1. Robinett CC*, et al.* In vivo localization of DNA sequences and visualization of large-scale chromatin organization using lac operator/repressor recognition. *J. Cell Biol.* **135**, 1685-1700 (1996).

2. Norrander J, Kempe T, Messing J. Construction of improved M13 vectors using oligodeoxynucleotide-directed mutagenesis. *Gene* **26**, 101-106 (1983).

3. Vlcek S, Korbei B, Foisner R. Distinct functions of the unique C terminus of LAP2alpha in cell proliferation and nuclear assembly. *J. Biol. Chem.* **277**, 18898-18907 (2002).
