# Supplementary material for: MyoD1 localization at the nuclear periphery is mediated by association of WFS1 with active enhancers
Source: Nat Commun. 2025 Mar 17;16:2614. doi: 10.1038/s41467-025-57758-x (PMC11914251; doi:10.1038/s41467-025-57758-x)
Supplement: Supplementary file 2 — Description of Additional Supplementary Files [file 41467_2025_57758_MOESM2_ESM.pdf]

Supplementary Movie 1

Description: 3D reconstruction of the nucleus of *MyoD1* LacO reporter cell line. Movie created with Imaris.

Supplementary Movie 2

Description: 3D reconstruction of the nucleus of *Pax7* LacO reporter cell line. Movie created with Imaris.
