# Supplementary material for: MyoD1 localization at the nuclear periphery is mediated by association of WFS1 with active enhancers
Source: Nat Commun. 2025 Mar 17;16:2614. doi: 10.1038/s41467-025-57758-x (PMC11914251; doi:10.1038/s41467-025-57758-x)

Supplementary Figure 1

c

***MyoD1* LacO**

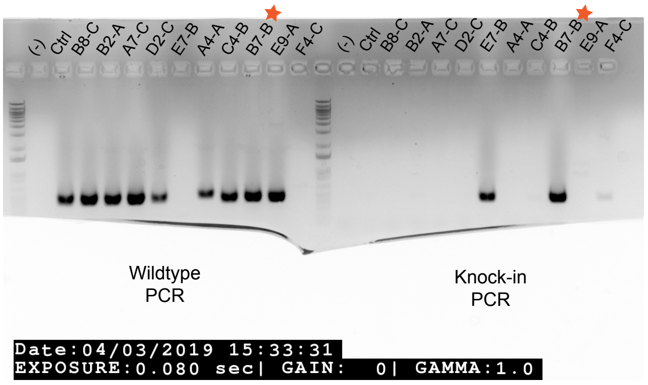

***Pax7* LacO**

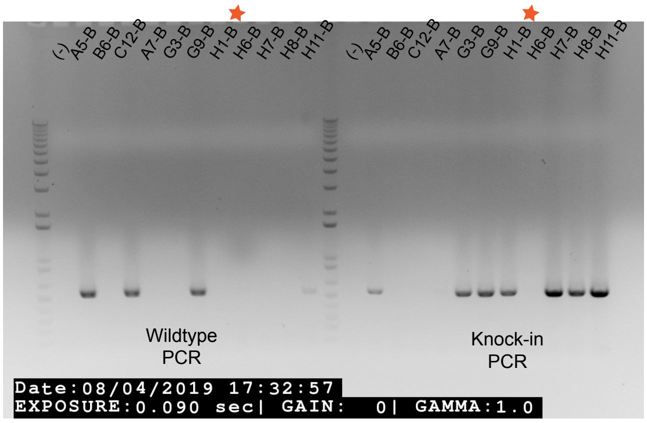

d

***MyoD1* LacO**

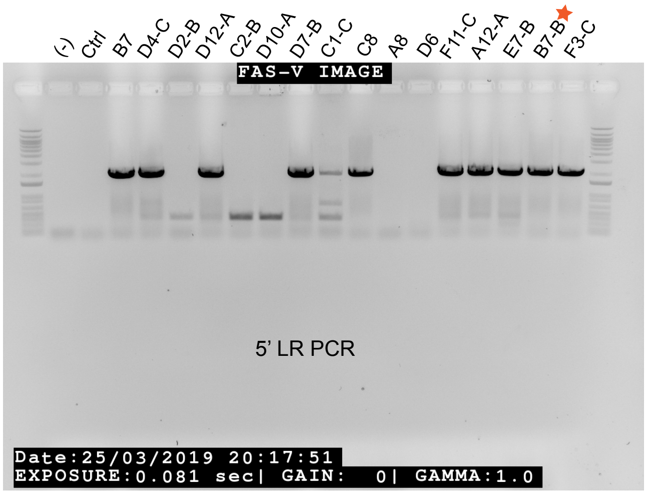

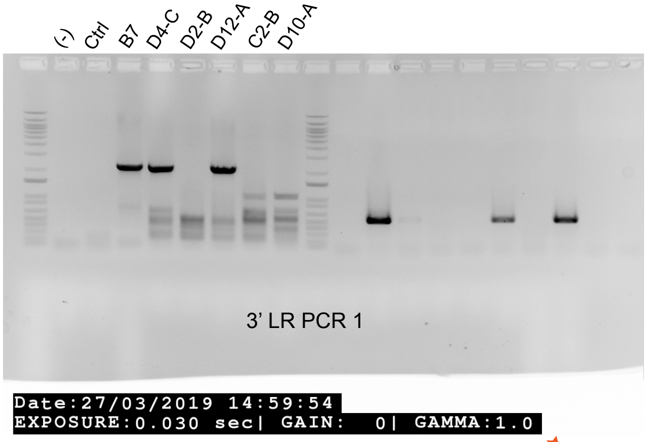

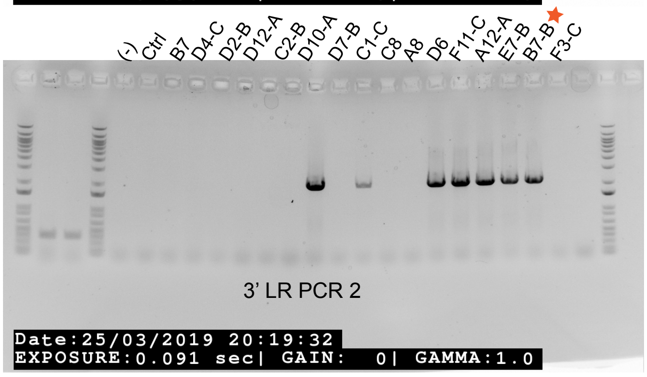

***Pax7* LacO**

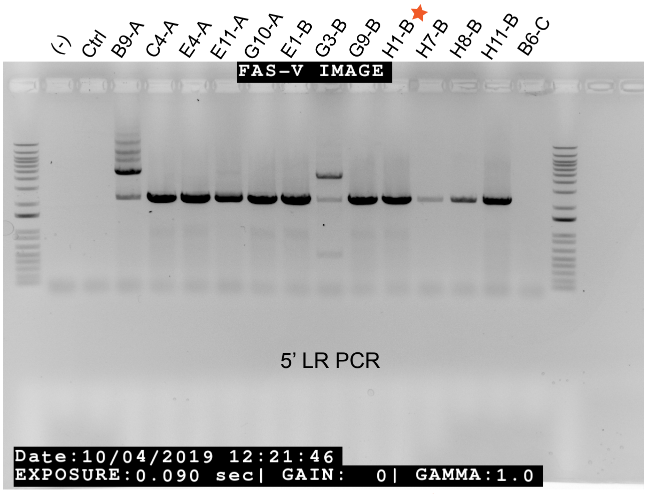

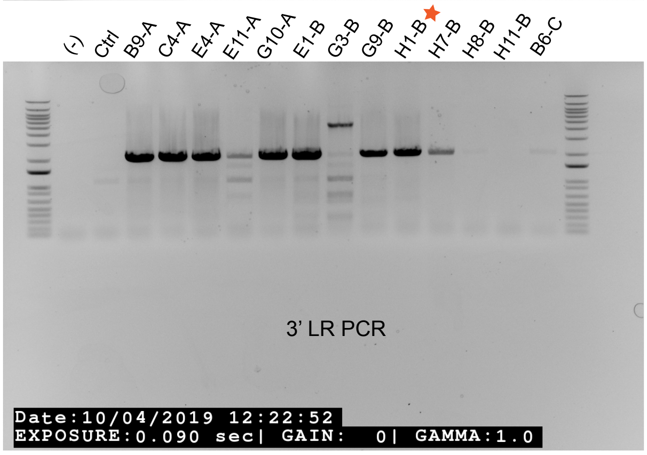

Supplementary Figure 1

h

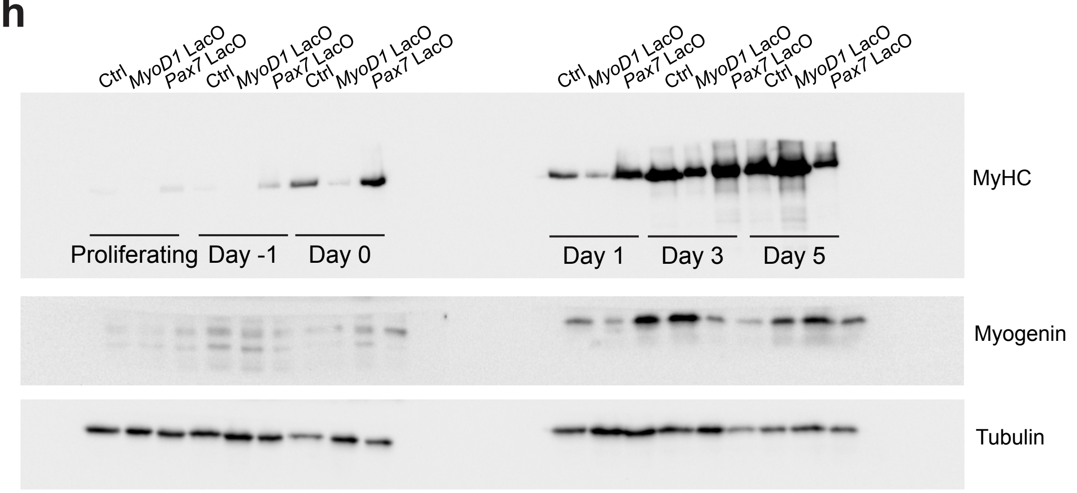

Supplementary Figure 3

a

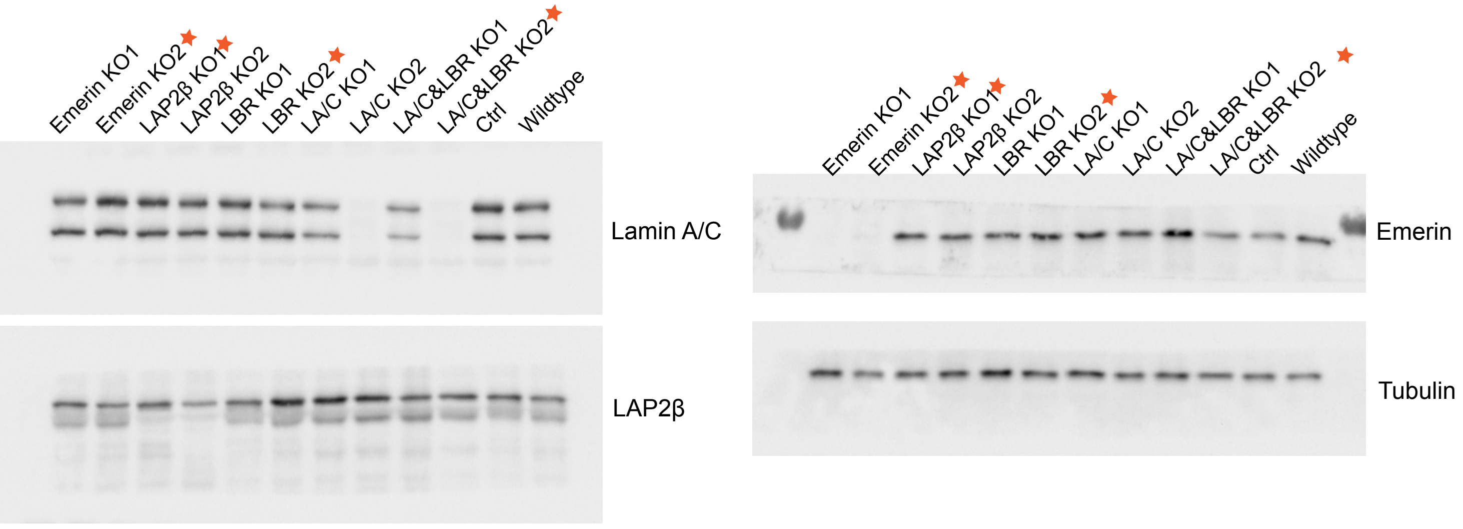

e

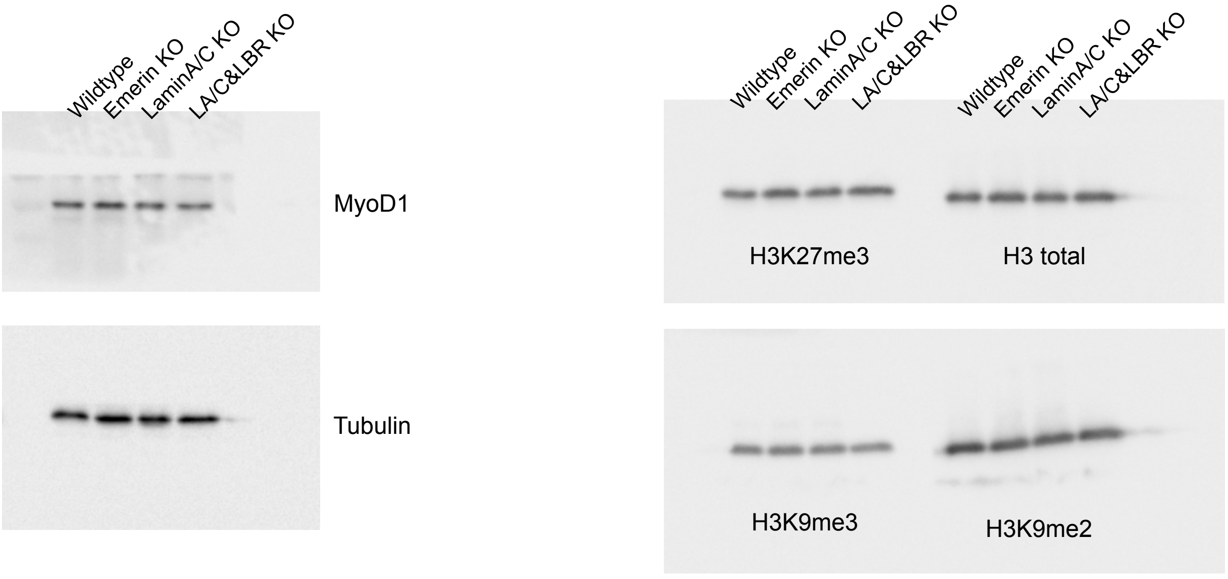

Supplementary Figure 3

h

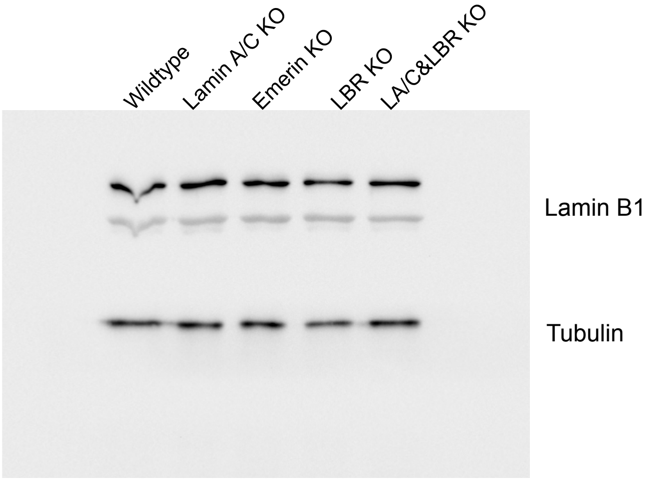

Supplementary Figure 4

b

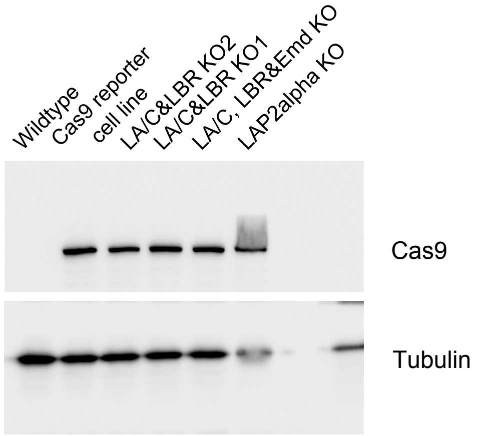

Supplementary Figure 5

e

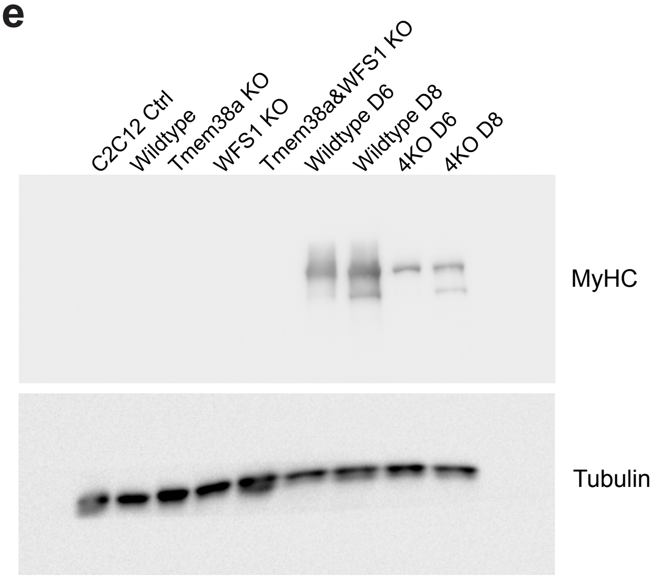

Supplementary Figure 6

c

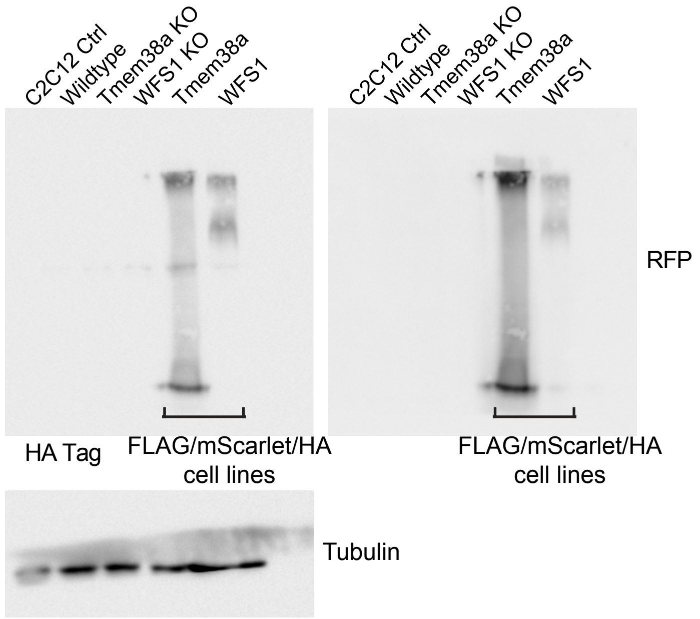

Supplementary Figure 6

d

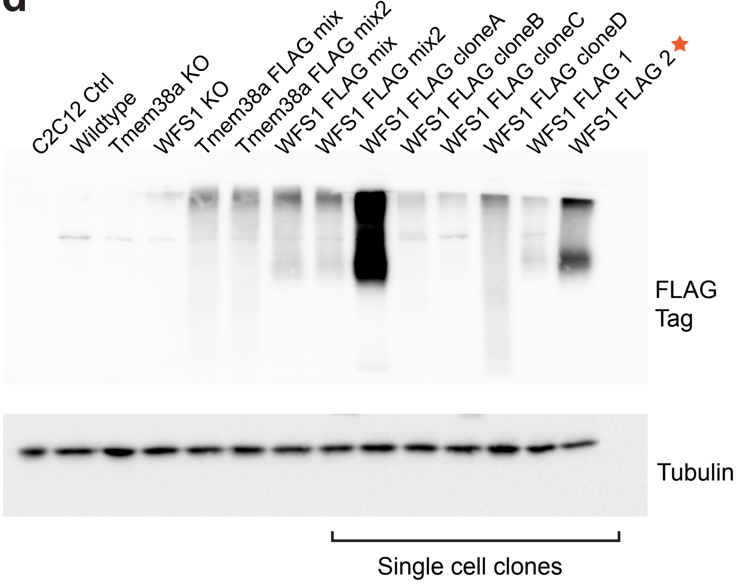

Supplementary Figure 7

a

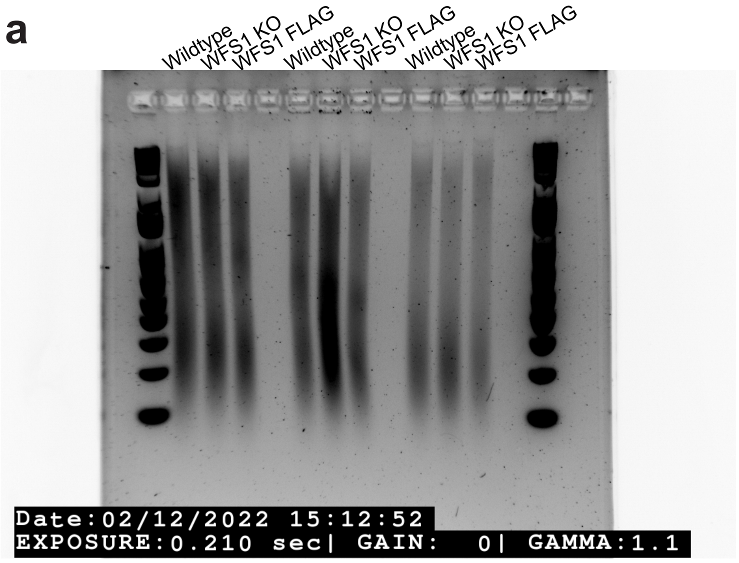

c

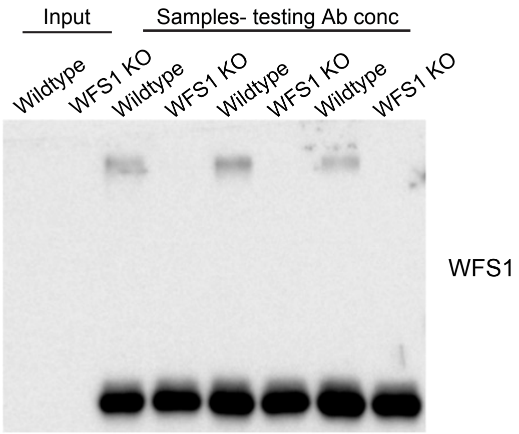

Supplement: Supplementary file 7 — Source Data [file 41467_2025_57758_MOESM7_ESM.zip › Source Data_Georgiou et al/Source Data_Supplementary Figures_gels.pdf]
